# At the Crossroads Between Psychiatry and Machine Learning: Insights Into Paradigms and Challenges for Clinical Applicability

**DOI:** 10.3389/fpsyt.2020.552262

**Published:** 2020-09-24

**Authors:** Sarah Itani, Mandy Rossignol

**Affiliations:** ^1^ Fund for Scientific Research (F.R.S.-FNRS), Brussels, Belgium; ^2^ Department of Mathematics and Operations Research, Faculty of Engineering, University of Mons, Mons, Belgium; ^3^ Department of Cognitive Psychology and Neuropsychology, Faculty of Psychology and Education, University of Mons, Mons, Belgium

**Keywords:** psychiatry, machine learning, diagnosis, open data, nosology and classification of mental**** disorders

## The Current Psychiatric Diagnosis: A Practice Subject to Debate

Good health and well-being feature among the development goals set by the United Nations members in their action plans to ensure peace and prosperity by 2030 (1). In this context, promoting mental health constitutes an important target. Additional efforts for an accurate, early and objective diagnosis of mental disorders can only contribute to move forward in this direction.

Classification systems—such as the Diagnostic and Statistical Manual of Mental Disorders (DSM), edited by the American Psychiatric Association (APA)—have been developed as a common language to conduct diagnosis in the most possible form of universality. However, these a-theoretical classifications lay down clinical descriptive criteria that can be open to subjective interpretation by clinicians. The APA tried to address this issue by fine-tuning the diagnostic criteria through the successive revisions of the DSM. However, each of these versions has always sparked lively debate in the community (2, 3). The explosion in diagnostic categories was notably heavily criticized. On the one hand, this multiplication was considered as a way of integrating the scientific progress of psychopathology research and offering more exhaustive descriptions (4). On the other hand, it has been argued that this explosion of diagnostic categories not only answers a commercial objective, but is also a way to satisfy the society’s tendency to organize and annotate the mental phenomena (5).

A diagnosis based on neuromarkers may respond to the criticisms addressed to the psychiatric classification systems (3, 6). Here, the greatest challenge remains to identify the relevant and discriminative markers that would reach sufficient scientific consensus.

## A Sense of Progress Through Machine Learning

Over the last decade, the application of Machine Learning (ML) to the psychiatric research has given the latter a new pulse (7). Indeed, in comparison to classical statistical approaches, ML provides outstanding capabilities for processing multivariate and multimodal data sets (8).

The ADHD-200 competition was probably the catalyst for inspiring such interdisciplinary research (9). This international contest was intended to accelerate the understanding of Attention Deficit Hyperactivity Disorder (ADHD), by inviting competitors to develop an imaging-based diagnostic classifier with the highest possible performance. The ADHD-200 collection is the first in a series of data sets released in the context of a large-scale project for open data sharing. This valuable culture was promoted by the 1000 Functional Connectomes Project (FCP), followed by the International Neuro-imaging Data sharing Initiative (INDI) (10). The will of opening research to the largest extent possible led to the sharing of software (11) and preprocessed data. The Autism Brain Imaging Data Exchange (ABIDE) is a notable example of the achievements of the INDI project. Related to Autism Spectrum Disorder (ASD), the ABIDE data set was released in two parts, including brain imaging data for over two thousand subjects aggregated across twenty-four worldwide imaging sites (12, 13). The analysis of such large data sets is expected to reduce inconsistency in research results, while the use of small sample sizes has demonstrated its limitations with variable levels of accuracy (14).

There is no doubt that the availability of large, free and well-structured databases has been a positive incentive for their analysis through ML, for the purpose of both knowledge extraction and diagnosis prediction. These capabilities were described in terms of their application to psychiatry (15–19). We will here discuss the main technical challenges of this ML-guided research.

## Toward a Successful Conjunction of Psychiatry and Machine Learning

### Designing Explainable Solutions

Over the years, the ML algorithms have been improved to be more and more performant. In particular, deep learning methods advantageously capture complex patterns in data, therefore allowing to reach higher levels of accuracy (20). But concretely, clinicians expect more than just high accuracy from predictive systems, which opacity constitutes a constant criticism (16, 19). The emerging domain of *explainable Artificial Intelligence* (xAI) is of particular interest in this respect (21). Indeed, explainability allows clinicians to choose to trust, or not, the recommendations (22). Moreover, it is well established that ML models tend to reproduce the biases present in the training data sets, often caused by the unbalanced representation of the classification categories. Explainable decision chains thus allow to control that the outputs are conform to ethical standards, and notably unsupported by any form of discrimination (23, 24).

Most of the attempts in developing an xAI were focused on the design of *post-hoc systems*, i.e. black boxes completed by a component explaining predictions a posteriori (25). Post-hoc systems are thus thought as an interesting way of combining high accuracy and explainability. However, they remain questionable on (i) the veracity of their explanations, which are generated around the considered data point, and (ii) the consequent inability to give a comprehensive picture of the model behavior (21).

Concurrently, it has been shown that models, ranging from white to black boxes, all perform comparatively when trained on quality and meaningful data (21, 26). This observation suggests a double perspective on the development of explainable ML systems.

- *Data preprocessing* conditions the performance of any decision system. This initial phase in the ML process can consist of applying a transformation to the original training features, in order to make them more discriminative. The transformation is sometimes unavoidably achieved through the (complex) combination of the initial training features, which introduces some interaction effects. Such a combination should thus be understood for the interpretation (even simplified) of the resulting features (27, 28).
- There also remain *theoretical challenges* to the improvement of the current predictive ML algorithms. A modern research avenue involves the design of optimal logical models (21) such as decision trees, that may be algorithmically strengthened to perform similarly as black boxes.

Efforts should thus be made both on data preprocessing and model design, in order to better address the need for explainability and transparency required by medical applications.

### Reconciling Theory and Data-Driven Approaches

Two main methodologies exist for scientific modeling (29).

- *Theory-based models*; that are grounded on known scientific laws based on some parameters, and a low amount of data is generally sufficient to fit these parameters.
- *Data-based models*; that require large data sets for an automatic training procedure which is expected to yield general models, able to describe the related phenomenon.

While theory-based methods are usually considered for the understanding of disorders, data-driven methods are rather considered for the design of clinical tools (16). A hybrid approach guided by data and theory would broaden the field of investigation, reconciling the existing scientific knowledge with elements extracted from data. The concept, which is not new, was highlighted in (16), and then properly formalized in (29) as the *Theory-Guided Data Science* (TGDS) paradigm. This principle should be encouraged in psychiatric research. Indeed, TGDS may be put in practice through the interaction with domain experts (i.e. psychiatrists, neuroscientists, neurologists) bringing their medical knowledge for feature selection (16), or more globally to refine ML models in the frame of an expert-in-the-loop mechanism (30). The aforementioned explainability naturally fosters the implementation of a TGDS.

### Considering One-Class Classification

Though they are mostly considered in the development of decision aid systems, Multi-Class Classification (MCC) algorithms are criticized for several reasons. Indeed, MCC does not address comorbidity appropriately since it considers the different diagnostic categories as mutually exclusive (16). In addition, MCC becomes more challenging in presence of unbalanced and noisy data sets (31). The domain of One-Class Classification (OCC) (32) covers a range of algorithms capable of describing a given class [e.g., a neuropathology (33, 34)], in such a way to reject cases that do not comply with this description. It is thus possible to use ensembles of one-class models in order to test a patient for several conditions simultaneously. One-class classification also gets rid of the need for a balanced data set including training instances from each class, as required by the MCC scheme. Finally, through its very nature, OCC can efficiently rule out noise, and specifically class noise which is usually located on the class boundaries (31, 35).

Several OCC tools are already available for clinical use, despite being mainly targeted towards neurological disorders (36). Hence, OCC deserves greater attention to be further developed in psychiatric research. Additional efforts for algorithmic improvements would be particularly worth considering in the context of explainable AI.

### Addressing the Question of Heterogeneity in Data

Though outstanding, the efforts for large-scale data gathering across several sites yield disparities in terms of demographics and experimental protocols (22). The homogenization of experimental protocols, and the design of appropriate validation procedures are respectively thought as ways for achieving and assessing generalizability (18, 19). The question of the extent to which this generalizability needs to be achieved deserves to be discussed, and probably requires a scientific consensus to provide a clear research direction. Indeed, the available financial and technical means for medical assessment and data may differ from a region to another.

Furthermore, psychiatric conditions can be characterized by clinical and/or neurobiological heterogeneity, the latter being also established in healthy controls (37). In this case, a thorough analysis may help to consider the best modeling strategy. For example, a ML framework can be implemented to perform diagnosis prediction in different levels, i.e., to detect a disorder first, and the disorder subtype then (38, 39). In (33), the authors focused on the description of ASD through OCC, since controls showed high neurobiological disparity. Moreover, ahead of the ML process, the experimental protocol should not necessarily be aligned with the DSM diagnostic categories. Indeed, these diagnostic labels are heterogeneous and derived from traditional assessments conducted by clinicians (40, 41). The Research Domain Criteria (RDoC) framework was introduced by the National Institute of Mental Health to alleviate this issue (42, 43). The RDoC orients the study of mental illnesses towards *domains of human functioning* described at different levels, rather than towards *symptoms*. The lowest level relates to *units of analysis*, suggesting relevant biological, genetic and physiological investigation markers (43).

### Encouraging Scientific Reproducibility

The psychiatric domain has witnessed a significant increase in ML-based studies, along with a diversification of the modalities considered for data processing and modeling (44). It is therefore imperative to apply guidelines that ensure reproducibility; recommendations are provided in (44). The appropriate choice of procedures for model training and evaluation, as well as the availability of source code/data should notably be encouraged. Yet, a recent study highlighted that these aspects are often lacking: it appeared that 50% of studies do not share software, while 36% do not give access to data (45).

More specifically in the context of open data sharing, a standard segmentation of the data collections into training and test sets would reinforce reproducibility (40). On the occasion of the ADHD-200 competition, training and test sets were kept separately in order to allow respectively the development and the assessment of the predictive models developed by the competing teams. Since then, these data subsets have mostly been used in their initial form, which makes it easy to report the evolution of the progress achieved on the prediction of ADHD. The same cannot be said for other INDI data sets such as the ABIDE collection, where the segmentation of data is a choice made for each research study. This great disparity in the definition of the data subsets therefore makes it difficult to track the research progress on a given mental disorder.

## Conclusion

Through the present perspective, we wished to draw attention on key principles for the design of Machine Learning (ML) solutions able to help clinicians to diagnose mental disorders. Our consideration addressed some main criticisms found in the literature about ML-based systems for clinical applications. It appears that a form of explainable and knowledge-guided data science will certainly help in the design of transparent mechanisms making sense to clinicians. The use of one-class classification algorithms allows to describe each neuropathological condition separately, and may better take into consideration comorbidity aspects. These practices are worth being encouraged, even though they are currently timidly implemented. Amid these capabilities, research will undoubtedly accelerate in addressing the question of heterogeneity in data and in encouraging scientific reproducibility. All these endeavors are definitely promising for the future of psychiatric research.

## Author Contributions 

Both authors contributed to the article and approved the submitted version.

## Funding

This work is funded by the Fund for Scientific Research (Fonds pour la Recherche Scientifique, F.R.S.-FNRS), Brussels (Belgium), through a research fellowship granted to SI.

## Conflict of Interest

The authors declare that the research was conducted in the absence of any commercial or financial relationships that could be construed as a potential conflict of interest.
